# A case report on over-replacement of oral calcium supplements causing acute pancreatitis

**DOI:** 10.1308/003588414X13824511650056

**Published:** 2014-01

**Authors:** V Pronisceva, J Sebastian, S Joseph, E Sharp

**Affiliations:** ^1^Dartford and Gravesham NHS Trust,UK; ^2^East Kent Hospitals University NHS Foundation Trust,UK

**Keywords:** Anaphylaxis, Patent blue

## Abstract

A 42-year-old female teetotaller presented via the accident and emergency department with a 2-day history of vomiting and upper abdominal pain. She was diagnosed with acute pancreatitis. The aetiology of the pancreatitis was identified as hypercalcaemia secondary to oral calcium supplementation. The hypercalcaemia was corrected by stopping calcium supplements and aggressive fluid resuscitation. A thorough literature search did not show any case reports in which the aetiology of pancreatitis was oral calcium supplement over-replacement.

## Case history

A 42-year-old female teetotaller presented via the accident and emergency department with a 2-day history of vomiting and upper abdominal pain. There was no history of diarrhoea or dysuria. On examination, she had epigastric tenderness, bowel sounds were present and there was no evidence of organomegaly. She was haemodynamically stable.

The patient’s past medical history included a total thyroidectomy (four years previously), post-thyroidectomy hypothyroidism and hypocalcaemia, peptic ulcer disease and epilepsy. Her drug history consisted of alfacalcidol (0.5μg, 2 times a day), calcium carbonate (1.5g [contains 600mg of calcium], 3 times a day), levothyroxine, omeprazole, topiramate, domperidone, phenytoin and amitriptyline.

Investigations showed: amylase 666u/l (normal range: 1–125u/l), calcium 4.9mmol/l (normal range: 2.2–2.6mmol/l), white cell count 13.6 × 10^9^ cells/l] (normal range: 4–11 × 10^9^ cells/l), creatinine 158umol/l (normal range: 49–90umol/l), urea 8.8mmol/l (normal range: 2.5–7.8mmol/l), random glucose 9.1mmol/l (normal range: 4.2–6.2mmol/l). Electrolytes, albumin, phosphate, liver function tests, lactate dehydrogenase, triglyceride, bicarbonate and C-reactive protein (CRP) were normal. Additional investigations showed: thyroid stimulating hormone 0.2miu/l (normal range: 0.4–5.0miu/l), free thyroxin 15pmol/l (normal range: 9–19pmol/l), parathyroid hormone 0.5pmol/l (normal range: 0.9–7.7pmol/l), vitamin D 73nmol/l (normal range: 50–140nmol/l). The pancreatitis was scored 0 using modified Glasgow score criteria. Chest x-ray was normal. Ultrasonography showed free fluid under the right hemidiaphragm. To rule out visceral perforation, contrast computed tomography (CT) of the abdomen and pelvis was requested. This showed signs of acute pancreatitis and peripancreatic fluid. The biliary tree was normal with no gallstones.

The aetiology of the patient’s pancreatitis was identified as hypercalcaemia secondary to oral calcium supplementation. The hypercalcaemia was corrected by stopping calcium supplements and aggressive fluid resuscitation. Hydrocortisone (200mg, single dose) was given the day after admission. Calcium levels came back to normal (2.6mmol/l, normal range: 2.2–2.6mmol/l).

The patient was managed conservatively with input and urine output monitoring. Owing to persistently elevated CRP levels, repeat CT was requested but showed no evidence of pancreatic necrosis. Ten days later she was discharged and restarted on a low dose of alfacalcidol (0.25μg, once a day) as per endocrinology advice. On four weeks’ review, the calcium levels remained normal. The patient is followed closely by an endocrinologist and her general practitioner.

## Discussion

Acute pancreatitis is a common acute surgical admission. The common causes of acute pancreatitis are ethanol abuse and gallstones. However, admission of acute pancreatitis secondary to hypercalcaemia is rare (drug induced acute pancreatitis incidence 1.4%).^1^ The most common drugs causing pancreatitis comprise didanosine, asparaginase, azathioprine, valproic acid and others.^1^ Causes of hypercalcaemia include primary/tertiary hyperparathyroidism, malignancy, granulomatous disease, renal failure, familial hypercalcaemic hypocalciuria, lithium associated hypercalcaemia, vitamin D intoxication and other causes.^2^ A thorough literature search did not show any case reports where the aetiology of pancreatitis was over-replacement of oral calcium supplements.

**Figure 1:**
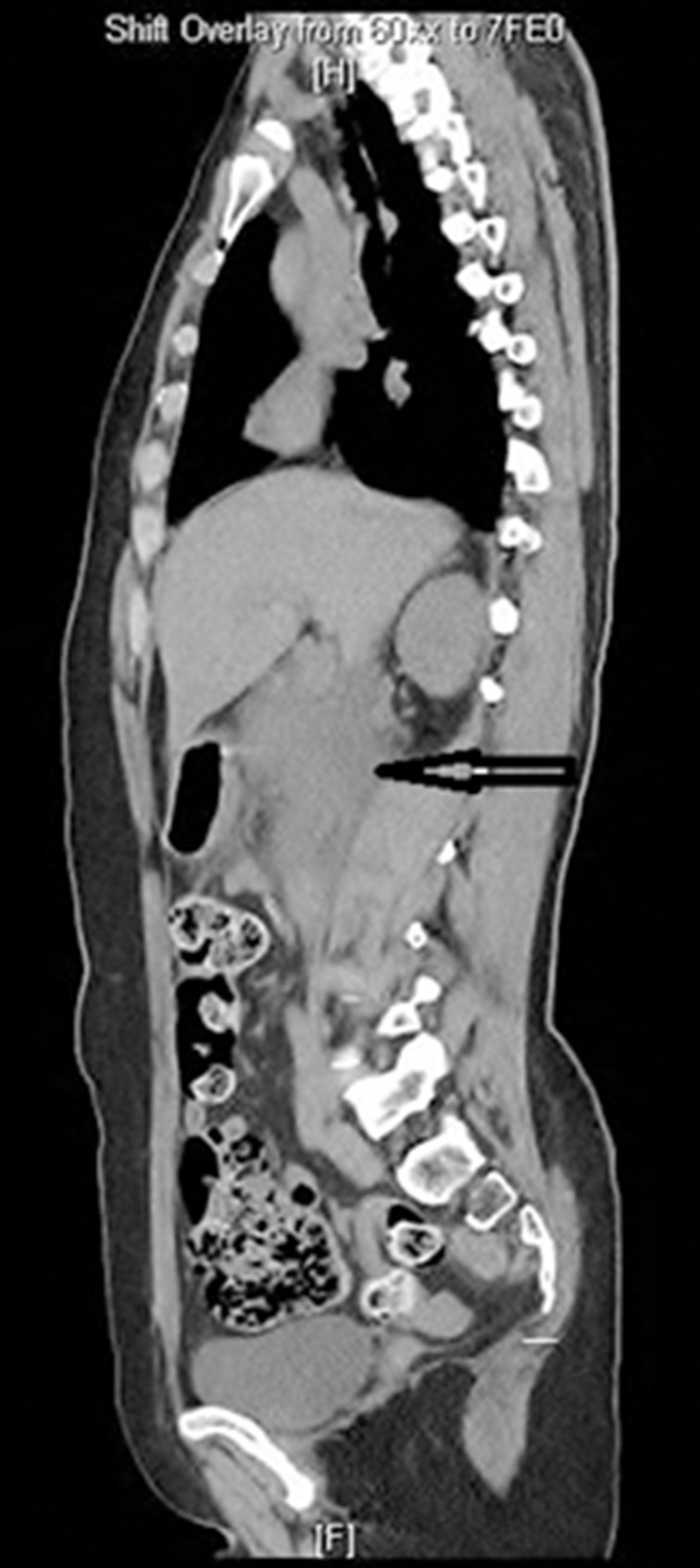
Sagittal axis computed tomography (CT) of the chest and abdomen on day of admission showing peripancreatic fluid in acute pancreatitis. The fluid extends into the right flank between the spleen and splenic flexure of the colon.

Three pathophysiological mechanisms have been suggested: 1) Deposits of calcium in the pancreatic duct may cause pancreatic duct obstruction. 2) Hypercalcaemia may lead to activation of trypsinogen in the pancreatic parenchyma, causing autodigestion of the pancreas. 3) Genetic variants in SPINK 1 and CFTR genes in combination with hypercalcaemia may also result in pancreatitis.^3^

The literature review showed several steps in hypercalcaemia correction but none of them applied specifically to hypercalcaemia in pancreatitis. It is well known that hypercalcaemia can be managed with aggressive fluid resuscitation and maintaining a high urine output. Urinary flow should exceed 250ml per hour during this time, and the serum calcium level will start decreasing within 2–4 hours and approach the normal range in 12–24 hours.^2,3^

This could be achieved by using an intravenous normal saline infusion (1–2l per hour), which will result in a marked increase in sodium, calcium and water delivery to the loop of Henle. In most of the papers, using furosemide 20–40mg intravenously every 2 hours is recommended although none of the authors commented about its use in pancreatitis. These actions will lead to a marked increase in urinary excretion of calcium but also of sodium, potassium, chloride, magnesium and water. They will therefore need monitoring and replacement, especially if this regimen is prolonged for longer than ten hours. Some authors mention benefits of steroids and bisphosphonates but steroid use in patients with severe pancreatitis is doubtful as it can worsen pancreatitis itself. Bisphosphonates have no immediate effect, taking 3–4 days to correct levels.^2^

The relationship between hypercalcaemia and acute pancreatitis remains controversial although several theories have been proposed based on pathogenesis.^4^ Among various forms of organ damage due to hypercalcaemia, acute pancreatitis is a rare but potentially lethal one. No specific guidance or recommendation on managing hypercalcaemia in pancreatitis seems to be mentioned in the literature.

## Conclusions

In our case, hypercalcaemia was diagnosed as a result of using the modified Glasgow score for pancreatitis but it was not suspected as a cause of the pancreatitis. This case emphasises the importance of scoring patients on admission. It encourages clinicians to keep in mind rare causes of pancreatitis. Regarding treatment, we would suggest concentrating on the causative agent of the acute pancreatitis as well as supportive treatment. In our opinion, oral calcium supplements can cause acute pancreatitis in patients following a thyroidectomy but this is extremely rare. We found no reports in our literature search.

We think that follow-up for patients with post-thyroidectomy hypocalcaemia on oral calcium supplements with vitamin D3 would be appropriate. However, the literature review did not reveal specific guidelines for the follow-up period. We suggest close follow-up for those patients who developed pancreatitis as a result of the hypercalcaemia as these cases have the potential to develop chronic pancreatitis and pancreatic insufficiency (based on pathogenesis theory).^4^ In addition, patients would benefit from joint care of surgical and medical teams (endocrinology). It is very important for junior doctors who come across patients with hypercalcaemia related pancreatitis to have knowledge and understanding of the pathophysiology as well as the skills to manage hypercalcaemia appropriately.

Based on evidence from the literature, patients with acute pancreatitis due to hypercalcaemia have an increased risk of developing necrotising pancreatitis.^5^ We therefore recommend early senior clinician input in management and treatment. In cases of concern (eg oliguria, hypotension, tachycardia), these patients would benefit from referral to the critical care team early, even if the initial pancreatitis score is 0.
